# Evidence for magnitude representations of social hierarchies: Size and distance effects

**DOI:** 10.1371/journal.pone.0203263

**Published:** 2018-09-07

**Authors:** Jostein Holmgren, Peder M. Isager, Thomas W. Schubert

**Affiliations:** Department of Psychology, University of Oslo, Oslo, Norway; University of Missouri Columbia, UNITED STATES

## Abstract

Social status is often metaphorically construed in terms of spatial relations such as height, size, and numerosity. This has led to the idea that social status might partially be represented by an analogue magnitude system, responsible for processing the magnitude of various physical and abstract dimensions. Accordingly, processing of social status should obey Weber’s law. We conducted three studies to investigate whether social status comparisons would indicate behavioral outcomes derived from Weber’s law: the *distance effect* and the *size effect*. Dependent variable was the latency of status comparisons for a variety of both learned and familiar hierarchies. As predicted and in line with previous findings, we observed a clear distance effect. However, the effect of size variation differed from the size effect hypothesized *a priori*, and an unexpected interaction between the two effects was observed. In conclusion, we provide a robust confirmation of previous observations of the distance effect in social status comparisons, but the shape of the size effect requires new theorizing.

## Introduction

Social status hierarchies are ubiquitous in human social life. We must recognize, encode, recall, update, and importantly compare individuals and groups regarding who is above whom. This informs us about who is likely to have resources and influences, who might prevail in negotiations and conflicts. Often these hierarchies include ourselves, or we must deal with individuals in them.

For all these social interactions, human cognition must facilitate social cognitions about hierarchies. Therefore, identifying the underlying cognitive system for understanding social status would give us a much better understanding of how humans interpret, understand and generate relationships. Several lines of research from social psychology and cognitive psychology converge to suggest that a special system exists for this type of social cognition, which handles hierarchical rank as *magnitude*. As in the existing literature on this topic, we use magnitude as a modality-neutral term for countable and uncountable quantities [1,2] on physical, numerical, and social dimensions. A key prediction of these accounts is that ranking decisions should show the signatures of magnitude processing, such as Weber’s Law. The current studies test this prediction for two signature effects.

### Hierarchical cognition

Relational Models Theory (RMT) suggests that hierarchical relations constitute one of only four different types of social relations for which humans are specifically prepared [3]. In social relations based on so-called *authority ranking*, individuals distribute resources according to a linear, asymmetrical ranking order [4]. For instance, a subordinate may pay tribute to an authority, while the authority offers protection over its subordinates. Examples of such authority ranking relations are a business, with a CEO, managers, and employees, or a university, with a president, deans, professors, and students. RMT has gained significant empirical support from a range of disciplines, including social cognition [5,6], neuroscience [7], psychopathology [8], and mathematics [4]. Assuming authority ranking constitutes one of only four possible social relation types, and a phylogenetically old one in addition, it is plausible that a distinct cognitive system may support this relation type.

People in authority ranking relationships often embody them using various kinds of magnitudes, and use physical metaphors to describe these relations [9]. A rank is labeled “high” or “low”, authorities are afforded more physical space, leaders are placed in front, and so on. Theories on embodied, or grounded, cognition argue that such correlations are often served by modal mental representations [10–12]. The underlying assumption of embodied cognition is that thoughts, emotions, and behavior are grounded in sensory and bodily states. The grounded cognition view proposes that reenactments of perceptual, motor, and introspective states are the content of information processing.

### Hierarchy as a magnitude

In line with the views that authority ranking is served by a dedicated cognitive module and the idea that such representations can be linked to systems that also serve cognizing concrete rather than abstract dimensions, recent work has argued that social hierarchies are represented as a magnitude, and are processed in the same cognitive system as physical magnitudes (e.g., [13–15]). This system, dubbed the analogue magnitude system, approximate magnitude system (AMS), or generalized magnitude system, was originally proposed to be a common mechanism for processing spatial, temporal, and quantitative information [2]. Evidence also exists supporting a common spatial and abstract numerical representation [16]. Assuming that social magnitudes indeed are represented in the AMS can elegantly explain both why we can derive hierarchies from dimensions such as bodily size, volume, and force, and why we have magnitude metaphors for hierarchy status that are rarely observed (e.g., brightness, [17]).

The intraparietal sulcus (IPS) is believed to be one important part of the neural correlates of the AMS (e.g., [2,18–20]). Recently, several studies have also linked social status comparisons to this brain region. Chiao and colleagues [21] found that military personnel comparing the status of military insignias and faces of military personnel to the midpoint of the dimension showed activation in the IPS bilaterally. The same was found for comparing Toyota car models to the midpoint of their status dimension. Cloutier, Ambady, Meagher, and Gabrieli found that self-referential financial, but not moral, status comparisons elicited increased activation in the IPS when the comparisons were implicit [22]. A subsequent study adding the explicit instruction to compare a target with oneself showed significantly increased IPS response during both financial and moral status comparisons [23]. Finally, Mason, Magee, and Fiske found increased activation in the left IPS during both body weight and status judgements (however, body weight judgements elicited significantly higher activation than status judgements in the left IPS, and the right IPS was only significantly activated by weight judgements) [24]. Taken together, these findings suggest that there may be a functional overlap in neurons in this region between physical, numerical, and social magnitude comparisons.

On the behavioral level, processing of physical stimuli is dependent on stimulus intensity as described by Weber’s law [25]. Weber’s law describes how an actual change in physical stimulus intensity relates to how that change is perceived by an observer. Weber’s law states that "Simple differential sensitivity is inversely proportional to the size of the components of the difference; relative differential sensitivity remains the same regardless of size." [26] Two distinct behavioral effects follow from Weber’s law. First, with increasing absolute difference in stimulus intensity in a stimulus pair, speed and accuracy of dissociation are improved. For example, discerning two weights of 2 and 5 kg (absolute difference of 3 kg) is easier than two weights of 2 and 3 kg (absolute difference of 1 kg). This is known as the *distance effect*. Second, with increased intensity of a stimulus pair, absolute difference kept constant, speed and accuracy of dissociation deteriorate. For example, discerning two weights of 1 and 2 kg is easier than two weights of 4 and 5 kg, even though the absolute difference is the same in both instances. This is known as the *size effect*. Alternatively, this can be conceptualized as manipulating the target intensity ratio through changing the magnitude of either or both targets. Both the size and distance effect are a product of how magnitude is mentally represented, but the observation of each effect has different implications for the nature of that underlying representation. The distance effect simply implies that a mental dimension exists on which units are placed in relation to each other. The size effect adds additional constraints to this representation, although several models with similar predictions have been proposed (particularly with respect to the domain of numerosity): 1) Magnitude is represented linearly, but with increasing variability (e.g., [1,27]), 2) magnitude is represented logarithmically with fixed variability (e.g., [28,29]).

Both the distance effect and the size effect have been found in reaction time studies of physical stimuli [30,31] and numbers [32–36] (see [16] for a review). Yet, only a few studies have so far demonstrated magnitude effects in social hierarchies, and all have focused on observing the distance effect.

Chiao and colleagues [37] presented a stimuli with social status connotations which had to be judged as lower, equal, or higher status than a reference midpoint. The stimuli were ranks of university occupations (Study 1) and military insignia (Study 2). Reaction times were averaged according to social distance from the midpoint of the hierarchy, which was used as the comparison standard. Only three categories (close, medium, far) were used. Longer distance resulted in faster judgments. Chiao and colleagues [21] employed a similar stimulus presentation, but the stimuli used were greyscale images of military insignia, faces of military officers in uniform, and cars, and only two levels of distance (close, far) were compared, with similar results.

Von Hecker and colleagues [38] presented names from fictional hierarchies of individuals in a vertical arrangement, and participants were tasked with selecting the higher or lower status individual (depending on condition). Again, higher distance results in faster reactions. Finally, Jiang and Zhu [39] presented individual power-connotated words (Study 1) and power-connotated word pairs in a horizontal arrangement (Study 2). The words used were not from any specific social hierarchy and were independently rated for power. Participants determined whether the word was low or high social status in absence of an explicit anchor (Study 1) and to select the higher status word (Study 2).

All these studies provided evidence for a magnitude effect such that comparison of status across more rank steps are facilitated. However, none of the studies provides a detailed picture of the distance effect in social hierarchies: Chiao and colleagues [21,37] averaged to broad categories, von Hecker and colleagues [38] tested at the same time the impact of vertical arrangement, potentially forcing a magnitude representation, and Jiang and Zhu [39] did not use a specific social hierarchy. Furthermore, no study has reported attempts to test for the size effect in social hierarchies.

The present article reports three studies investigating both the distance and the size effect in social status decisions. In Study 1, novel social hierarchies were taught to participants prior to a binary choice task. Study 1 is a partial replication of a study reported by von Hecker et al. [38]. In Study 2 and 3, rank labels from existing hierarchies were used in the same binary choice task, with six and seven levels, respectively. We thus test both newly learned and well-known hierarchies with a large number of levels that we can look at separately.

In this series of studies, we attempt to replicate previous findings of the distance effect of social magnitudes with greater resolution across the levels for specific hierarchies and without additional primes of magnitude representations. We also test whether a size effect can be observed. Regarding the distance effect, we hypothesized that in a linear social hierarchy structure, deciding who is of higher rank would be increasingly faster and more accurate with increased distance (H_1_). Regarding the size effect, we hypothesized that deciding who is of higher rank would be faster and more accurate the lower in the hierarchy the lower comparison target is ranked (H_2_).

All studies were approved by the internal review board of the Department of Psychology at University of Oslo, and conducted in a laboratory at the department. Written informed consent was obtained from all participants prior to their participation. No formal power analyses were conducted. Sample size was set based on the sample size of previous behavioral studies investigating the distance effect in social magnitudes [21,38]. Results were only analyzed after the previously determined sample size was reached. All data, analyses, and materials are available from the Open Science Framework at https://osf.io/t45zd/.

## Study 1

In Study 1, we tested distance and size effects in newly learned hierarchies with six levels. Participants learned six social hierarchies of individuals through stories. After each story, a series of trials presented two members of the hierarchy. Participants decided which one had higher status (or lower status, manipulated between participants).

### Method

#### Participants

Thirty students (16 female, mean age 22.5, *SD* = 2.3) at the University of Oslo participated in the experiment. Some participated as part of an obligatory course requirement, with the option of withdrawing consent at any point without being penalized. The rest were recruited from the university campus on a voluntary basis.

#### Materials

Six stories were used to teach the participants six status hierarchies. The stories were based on Von Hecker and colleagues’ [38] materials (translated and adapted from German to Norwegian). Each story consisted of 16 sentences. The first sentence in each story was always an introduction to the topic of the story. The following 15 sentences in each story illustrated the status of six persons in each story referred to by name. Each sentence described an interaction between two of the people in the story, corresponding to the 15 possible pairings between the six persons. The names for each story were randomly selected for each participant out of a pool of 42 names of approximately equal popularity and without a priori social status implications.

#### Procedure

Participants read each story sentence by sentence on a computer screen, using the spacebar to proceed when they were ready. The first sentence was always the same for all participants, presenting the topic of each story. The other 15 sentences were presented in randomized order. The participants were encouraged to think about the status implication between the persons in each story as they read. After each story, the participants were presented with trials where they had to make forced-choice decisions about the status of two individuals presented in the story.

Each trial started with a fixation cross (750 ms), followed by pairs of names side by side from the story they just read. The participants were to select the person with higher or lower status, depending on the condition, by pressing on a keyboard the “a” key to select the left target or the “l” key to select the right target. In each trial, participants had five seconds to respond, automatically proceeding to a blank screen (500 ms) followed by the next trial if no response was given. The procedure was programmed in E-Prime 2 and presented on 17” flatscreen monitors, using black font on a white background.

All 15 possible pairings were presented in the two possible configurations for each (AB and BA). After one block of these 30 configurations had been presented, the block was repeated, resulting in a total of 60 trials in a block corresponding to each story. Order of trials within blocks was randomized, as was order of stories (hierarchies). At the end of the experiment, information about each subject (age, gender, etc.) was collected. The experiment lasted about 45 minutes debriefing included.

### Results

Data were analyzed using a linear mixed model [40]. We employed MIXED in IBM SPSS 24.0. Log-transformed individual reaction times of correct answers were chosen as the units of analysis and basis of statistical tests reported here in order to meet the assumption of normal distribution of dependent variable values. Averages of untransformed latencies are shown in graphs and tables for ease of interpretation. The analysis design was 5 (Distance) x 5 (Height) x 6 (Story) x 6 (Block Order) x 2 (Search Condition). All interactions were included. Distance was defined as the relative difference in rank between the highest and the lowest in a pair (6 levels, so max distance was 6–1 = 5, minimal distance was 1). Height was defined as the rank of the lower-status member of each pair, in accordance with previous definitions (e.g., [32,34]). Note that not all cells of this design are filled—e.g., there cannot be a combination of distance = 5 and height = 4. However, the use of mixed models allows us to estimate the parameters with this incomplete design.

A participant ID was added as a random factor, letting intercepts vary across it. In initial models, Story was added as a random factor as well instead of as a fixed factor, again letting intercepts vary across it. However, it explained very little random variance, and caused errors in the model estimation (Hessian matrix was not positive definite), and was therefore removed from the analysis. Also, additional analyses confirmed that the findings for the main effects hold when the different stories are tested separately.

All incorrect answers were excluded, as we only predict that the specified hypotheses are valid when a correct judgement has been made. It is routine procedure to exclude participants with an extreme rate of incorrect responses, but the criteria for cut-off is highly dependent on the specific experimental paradigm [41,42]. If a participant answered incorrectly over 20% of trials for any block, then all trials from that block were excluded for that participant. Von Hecker and colleagues [38], whose paradigm is comparable to our own, reported error rates of 13.6% and 16.8% for their two studies. We elected to exclude individual blocks exceeding the threshold rather than whole participants, because we could not assume all blocks would be equally difficult. However, if more than 50% of blocks were excluded for any participant, all trials from that participant were excluded. No blocks or participants were excluded by these criteria in Study 1. Due to the nature of the task, for all response times above 5000 ms we assumed that the subject had become distracted or made an error (e.g. pressed the wrong key). The response latency distribution was normalized through log transformation. For all response times below 400 ms we assumed that the subject did not have sufficient time to read and compare both words. These response times were all excluded. These criteria are in line with typical treatment of response latencies [41,42] (unless errors are explicitly modelled as well) and were determined before data analysis took place. In total, 10.2% of trials were excluded.

**Fig 1:**
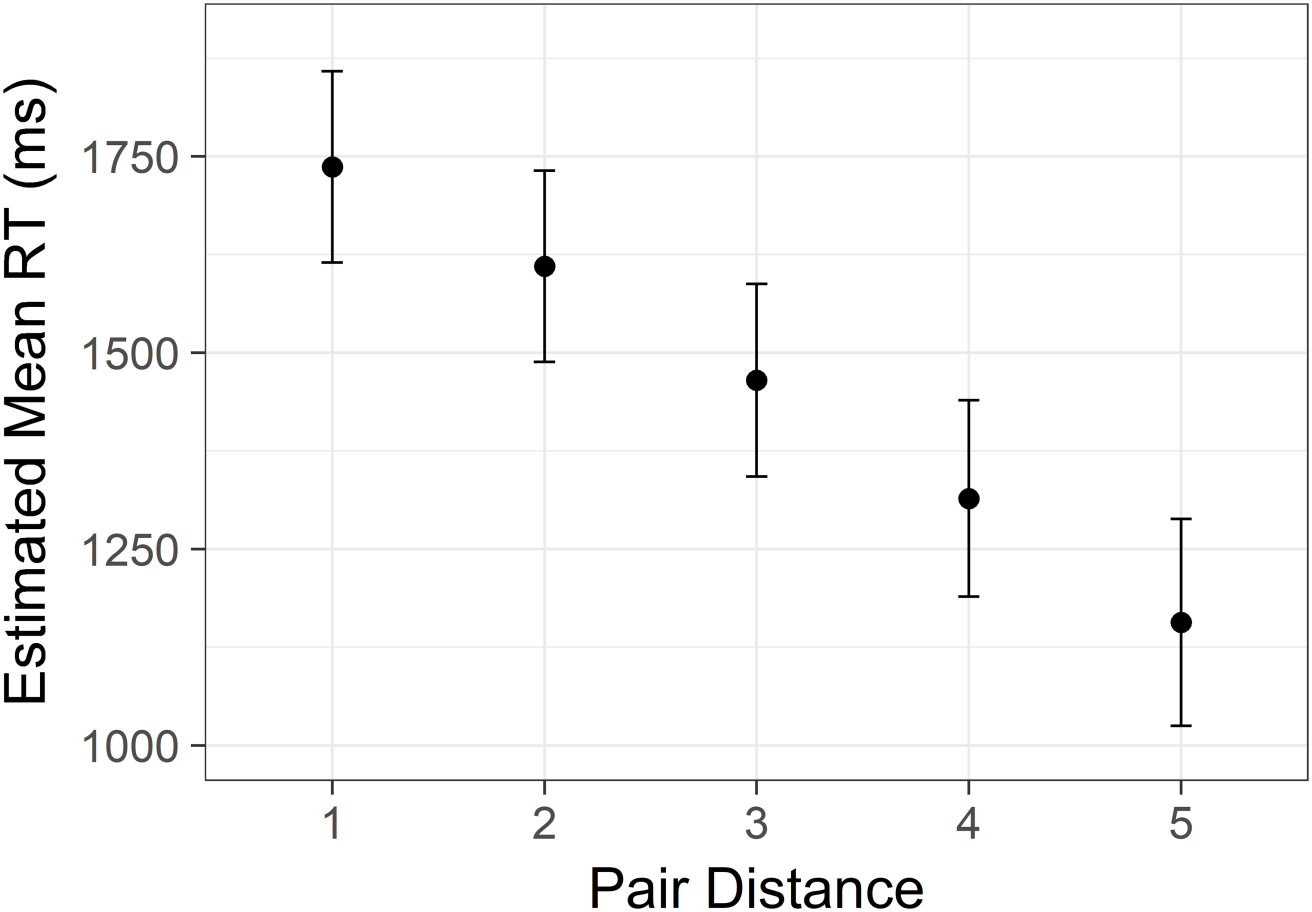
Linear distance effect from Study 1. Estimated mean time spent correctly identifying the highest/lowest status target in a pair with increasing difference in status between the targets. Y axis is scaled in milliseconds. Error bars represent 95% confidence intervals.

**Fig 2:**
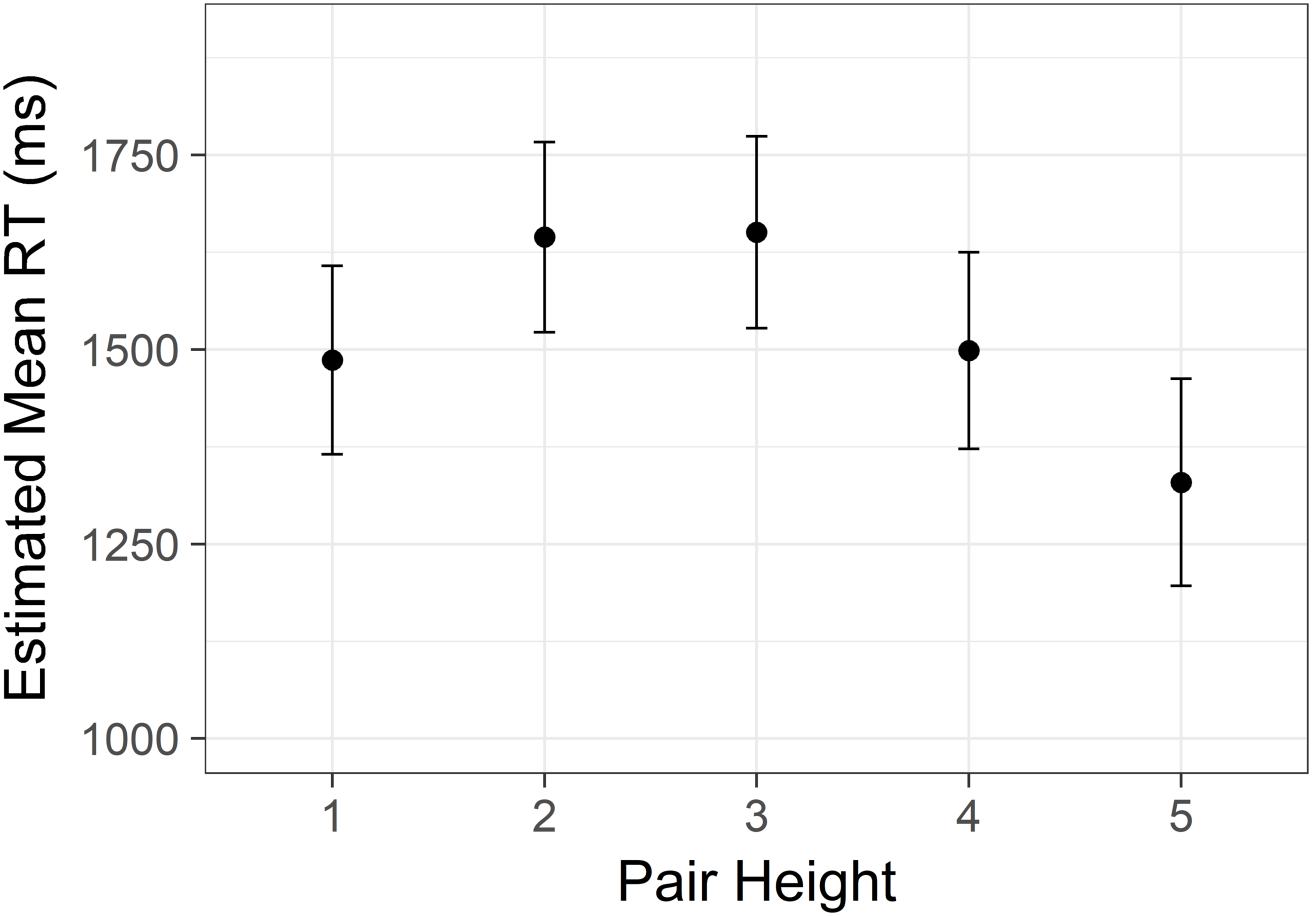
Non-linear size effect from Study 1. Estimated mean time spent correctly identifying the highest/lowest status target in a pair with increasing status of the lower member of the target pair. Y axis is scaled in milliseconds. Error bars represent 95% confidence intervals.

There was a significant main effect of distance *F*(4, 7426) = 197.79, *p* < .001. Pairwise comparisons revealed a clear linear decrease in reaction time with each step of increasing status distance (all Sidak corrected *p*s < .001, Fig 1). There was also a significant main effect of height *F*(4,7427) = 100.07, *p* < .001. This effect was clearly non-linear. These two main effects are graphed in Figs 1 and 2. Note that we display confidence intervals to convey information about the distribution, but because the key comparisons are within-subjects, the tests reported here are more informative.

**Fig 3:**
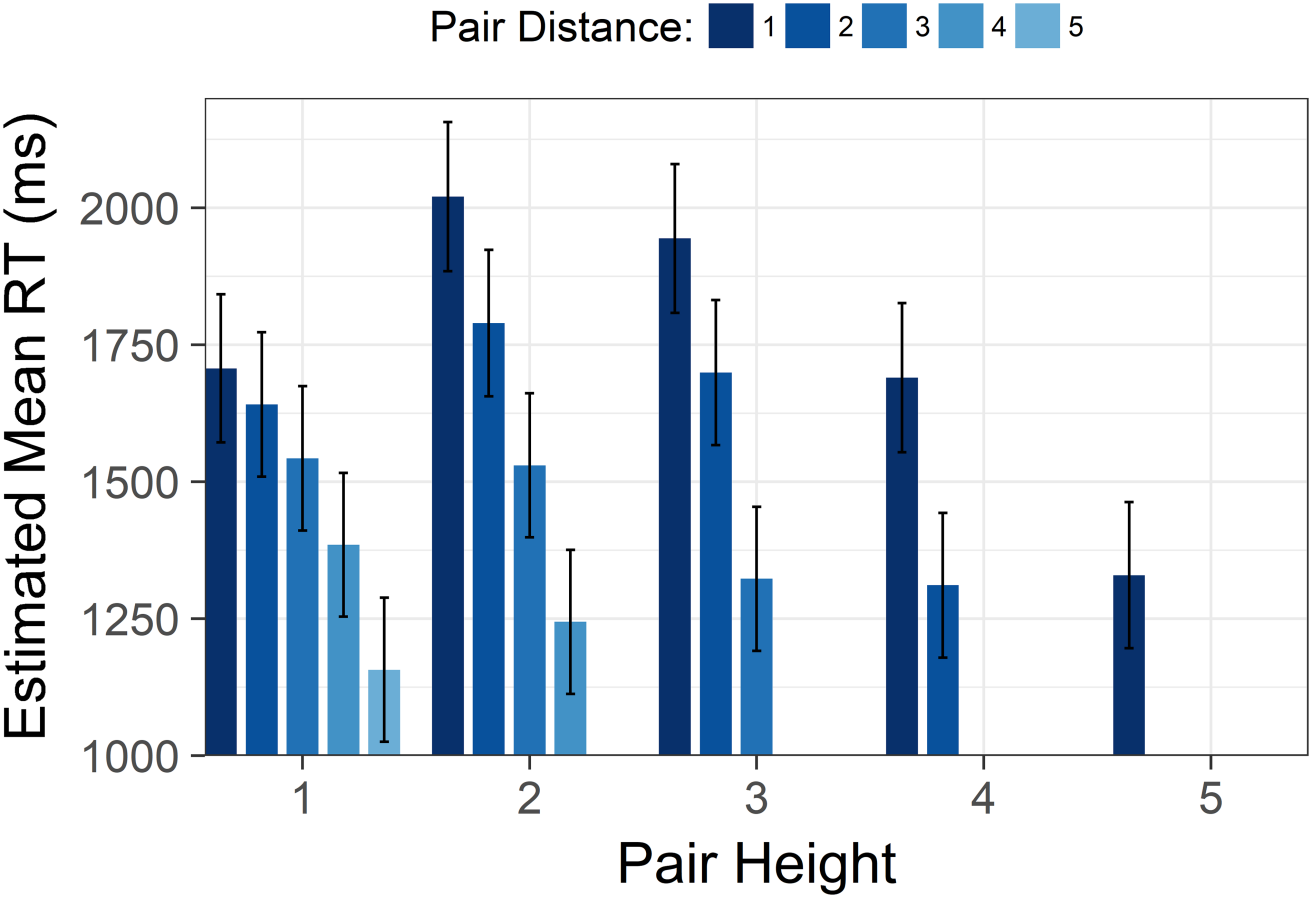
Size by distance interaction from Study 1. Estimated mean time spent correctly identifying the highest/lowest status target in a pair with increasing status of the lower member of the target pair. Each cluster displays the estimated means with increasing pair distance for that level of height. Y axis is scaled in milliseconds. Error bars represent 95% confidence intervals.

Furthermore, we found an interaction between distance and height, *F*(6,7426) = 23.29, *p* < .001. The interaction indicated that the effect of increasing distance grew more pronounced as comparisons were made higher in a hierarchy (Fig 3).

In addition, there were several other significant interactions, all explaining much less variance. Randomization and counterbalance procedures in the design ensured that these effects did not bias the hypothesis-driven tests for height and distance. Our explorations of these moderations did not show any substantial modulation of our focal effects. They will not be further detailed here (see S1 Table for a full overview of the fixed effects from the mixed models analysis).

A potential experimental artifact in all the studies is that subjects knew the end-points of the hierarchy in each story. They could therefore immediately respond correctly whenever they saw a stimulus constituting the highest or the lowest status of a hierarchy, without needing to look at the other stimulus in the pair. To safeguard against this potential artifact, we reran all analyses while removing all comparisons containing end points. For the height and distance effects, the shape and significance of the effects remained the same in this analysis. The interaction was no longer significant. However, it is difficult to compare these analyses because less than half of the original pair-wise differences were available for comparison after end-point exclusion, and removing two out of six hierarchy levels constitutes a 33% decrease in range of the independent variables.

### Discussion

Study 1 replicated earlier findings from von Hecker et al. [38]. Latencies for making decisions about who is higher (or lower) in newly learned hierarchies show a clear distance effect. The further apart the judged individuals are in the hierarchy, the faster the decision. This is in line with predictions from understanding hierarchy representations as part of the magnitude system. Note that it contradicts the intuitive notion that one should be more knowledgeable about immediate pairings.

However, the effect of stimulus-pair size, or more precisely the rank of the lower individual in the compared pair, did not follow predictions from the magnitude system. We had expected that the further up in the hierarchy comparisons were made, the slower reactions should get. Instead, we observed a reverse U-shape, with fast reactions for trials where the lower individual was either very low or very high in the hierarchy.

## Study 2

In Study 2, we investigated whether the results from Study 1 would replicate if the story-learning paradigm was substituted by status levels from familiar hierarchies. Study 2 also employed hierarchies with six levels.

### Methods

#### Participants

Thirty students from the University of Oslo, and Oslo and Akershus University College of Applied Sciences, (18 female, mean age = 22.9, *SD* = 2.5) took part in the experiment. Some participated as part of an obligatory course requirement, with the option of withdrawing consent at any point without being penalized. The rest were recruited from the university campus on a voluntary basis.

#### Material

Three social hierarchies, presented in written form, were used. One consisted of Catholic religious ranks, one of feudal political ranks, and one of military ranks. Each hierarchy contained six status levels, all intended to be familiar to the participants, and all intended to be related to all the other levels on the same hierarchy (see Fig 1).

#### Procedure

All participants were presented with every hierarchy, one at a time. This was done after acknowledging that no hierarchy would be equally familiar to all participants. Before the actual task, each hierarchy was presented on the screen in vertical linear order and participants were given as much time as they wanted to memorize them.

After seeing the hierarchy, participants completed 30 practice trials with, and then 60 experimental trials without feedback. The feedback consisted of a screen displayed for 2 seconds after each practice trial, telling the participant if the answer was correct, the percentage of trials he/she had answered correct up until that point, and how fast he/she responded. Instead of hard-coding a 5000 ms response window as in Study 1, we opted to give participants unlimited time to respond in Study 2 to minimize interfering with their task. We then applied the same 5000 ms window at the analysis stage. The task was otherwise the same as in Study 1 with levels from the presented hierarchy replacing names from stories.

At the end of the experiment, information about each subject (age, gender, etc.) was collected. The experiment lasted about 30 minutes, debriefing included.

### Results

Data was subjected to a linear mixed model analysis. The design was 5 (Distance) x 5 (Height) x 3 (Presentation Order) x 2 (Search Condition) x 3 (Hierarchy type). All interactions were included. Exclusion criteria and unit of analysis were the same as for Study 1, substituting hierarchy for story. One full block, but no participants, was excluded due to exceeding the error rate threshold. In total, 8.25% of trials were excluded.

**Fig 4:**
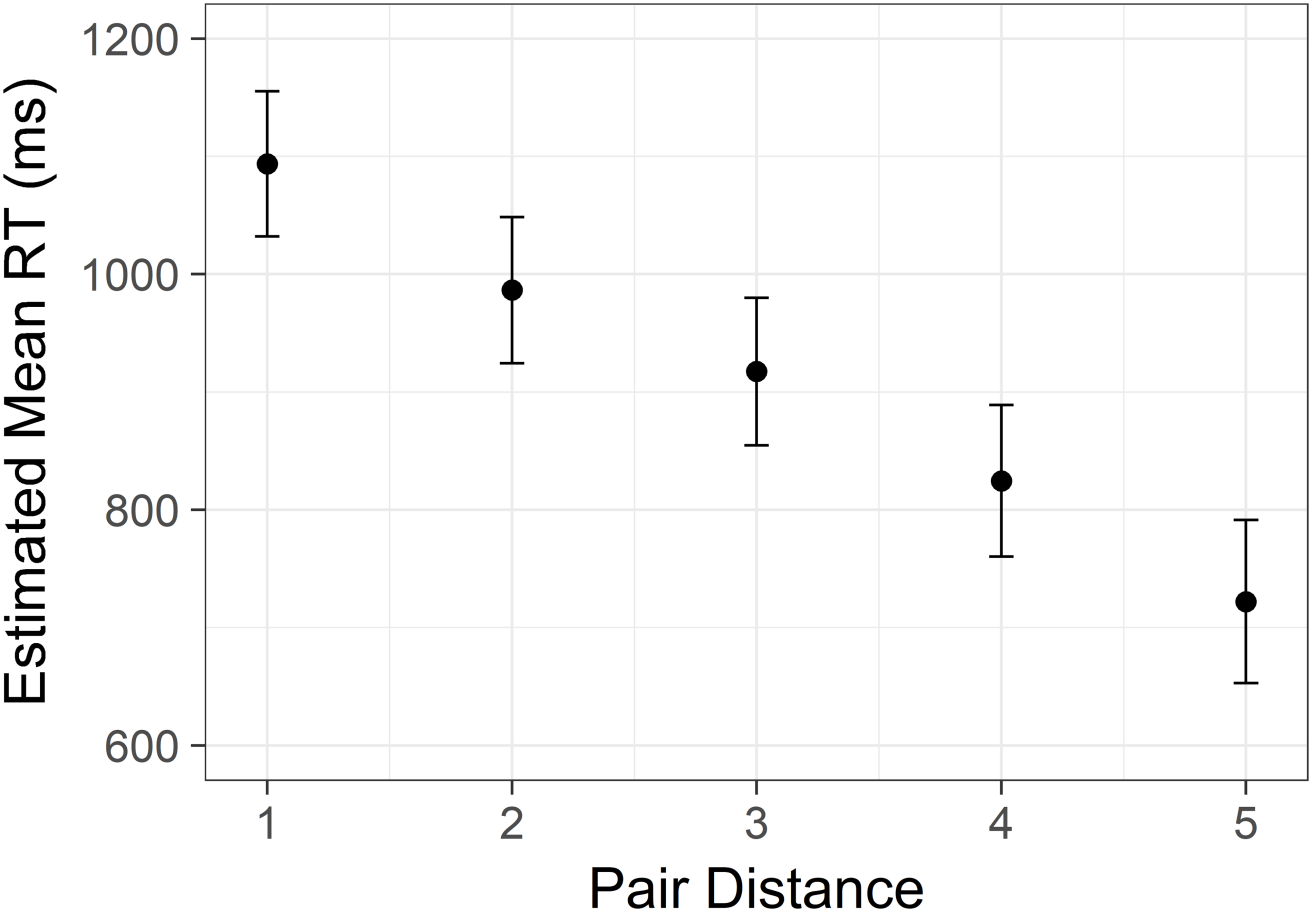
Linear distance effect from Study 2. Estimated mean time spent correctly identifying the highest/lowest status target in a pair with increasing difference in status between the targets. Y axis is scaled in milliseconds. Error bars represent 95% confidence intervals.

There was a significant effect of distance *F*(4,4525) = 150.39, *p* < .001. As in Study 1, there was a clear linear decrease in reaction time for increases in distance (Fig 4), all pairwise comparisons were significant (Sidak corrected, all *p*s < .001). The effect remained significant after end points were excluded from the data.

**Fig 5:**
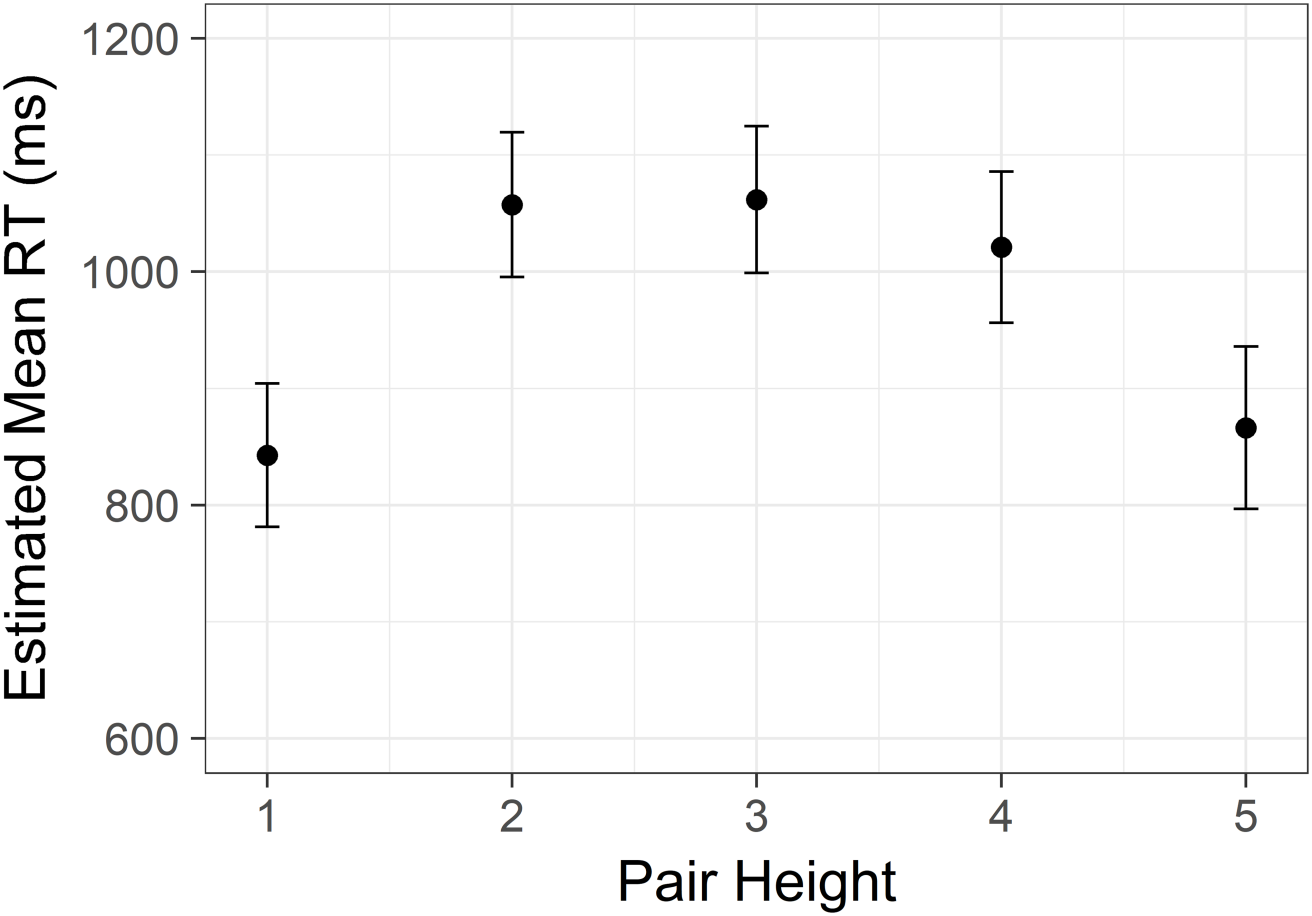
Non-linear size effect from Study 2. Estimated mean time spent correctly identifying the highest/lowest status target in a pair with increasing status of the lower member of the target pair. Y axis is scaled in milliseconds. Error bars represent 95% confidence intervals.

There was a significant effect of height *F*(4,4525) = 110.75, *p* < .001, with estimated means resembling the pattern in Study 1 (Fig 5). The effect remained significant after end points were excluded from the data in additional analyses.

**Fig 6:**
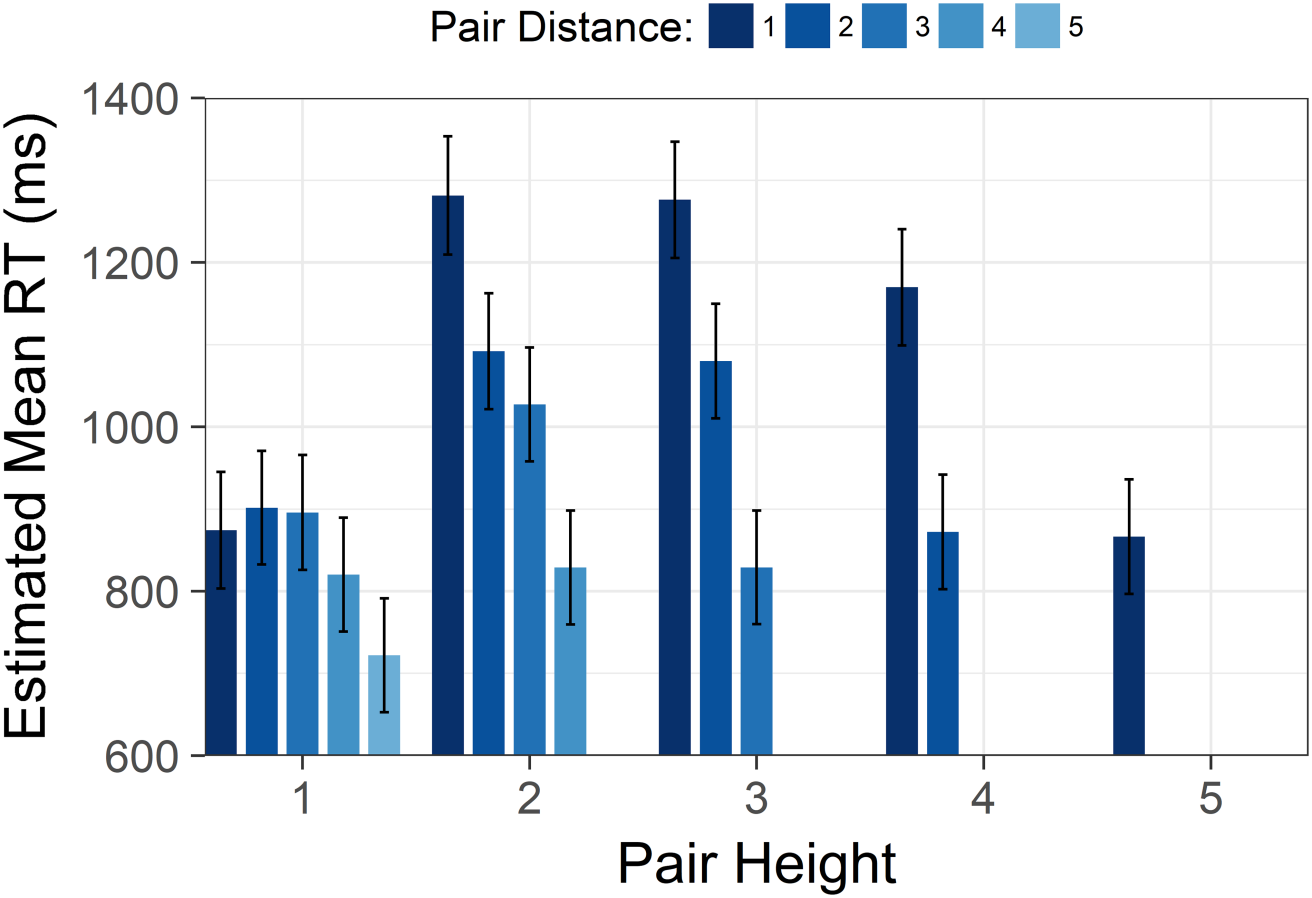
Size by distance interaction from Study 2. Estimated mean time spent correctly identifying the highest/lowest status target in a pair with increasing status of the lower member of the target pair. Each cluster displays the estimated means with increasing pair distance for that level of height. Y axis is scaled in milliseconds. Error bars represent 95% confidence intervals.

As in Study 1, there was a significant interaction between height and distance, *F*(6,4525) = 52.66, *p* < .001. The shape of the interaction was highly similar to the interaction in Study 1 (see Fig 6).

Several other main effects and interactions were significant but are not described here for the same reasons as stated in Study 1. A full overview of fixed effects can be found in S1 Table.

### Discussion

Deciding who is higher (or lower) in a known hierarchy shows the same predicted shape of the distance effect and the same unexpected shape of the size effect as newly learned hierarchies.

We suspected that the inverted U-shape in both studies could be an experimental artefact caused by the hierarchies having defined end-points known to the participants. It is possible that participants adopted a strategy where they would omit comparing the targets in trials containing one or both of the highest and lowest possible targets, knowing that those targets would always warrant the same response (e.g. if tasked with selecting the highest target, king would always be the correct target). This would allow for faster responses for these trials, potentially causing the inverted U-shape at each end of the hierarchy.

To control for this experimental artefact, we would have to exclude all trials containing either end-point. However, with only six levels in each hierarchy this would leave only 40% of the original number of trials. In addition, this would reduce the range of ranks to 4, allowing only two pairwise comparisons (between height 2 to 3 and height 3 to four). We therefore conducted a third study with expanded hierarchies to address this potential artefact.

## Study 3

Excluding end-points from the analysis in Studies 1 and 2 left only four valid status levels and excluded the majority of trials. To retain enough levels for a meaningful analysis while excluding hierarchy end-points, we replicated the design of Study 2, adding one more level to each hierarchy, and removing the military hierarchy.

### Method

#### Participants

Fifty-two students from the University of Oslo, and Oslo and Akershus University College of Applied Sciences, took part in the experiment (32 female, mean age = 22.7, *SD* = 4.7). Some participated as part of an obligatory course requirement, with the option of withdrawing consent at any point without being penalized. The rest were recruited from the university campus on a voluntary basis.

#### Materials and procedure

We included one additional level for the feudal and the Catholic religious hierarchy. We removed the military hierarchy to keep the total experiment duration comparable to that of Study 2, in particular to limit fatigue reducing the quality of later trials. The military hierarchy was removed because participants in Study 2 consistently reported being least familiar with this hierarchy. This left two hierarchies with 21 unique status comparisons for each. Besides this, materials and procedure were identical to Study 2.

### Results

Data analysis was conducted in the same manner as Study 2. The only difference was that the height and distance factors now had 6 levels each. One full block, but no participants, was excluded due to exceeding the error rate threshold. In total, 5.7% of trials were excluded.

**Fig 7:**
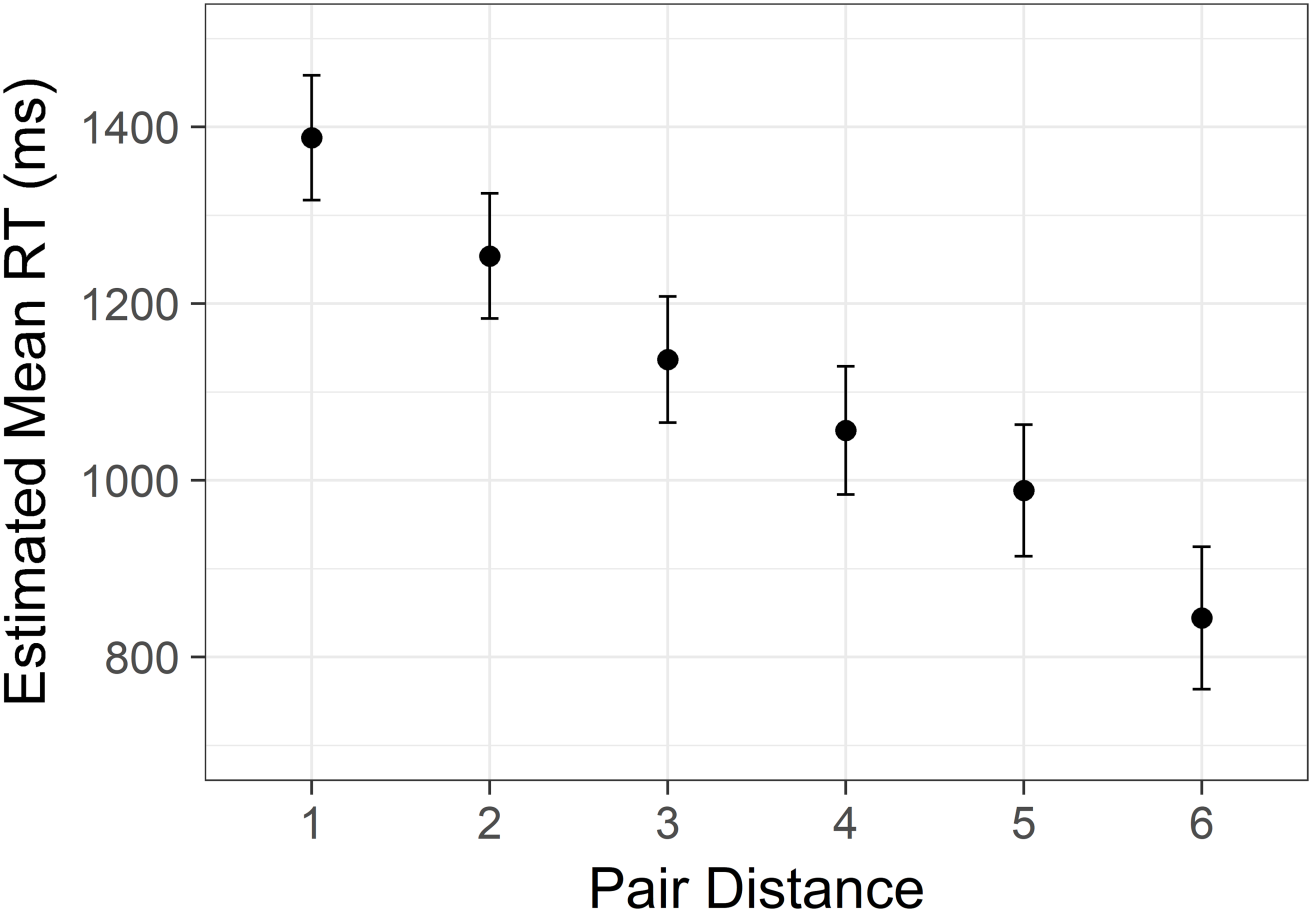
Linear distance effect from Study 3. Estimated mean time spent correctly identifying the highest/lowest status target in a pair with increasing difference in status between the targets. Y axis is scaled in milliseconds. Error bars represent 95% confidence intervals.

There was a significant effect of distance *F*(5,8338) = 306.02, *p* < .001, similar to that of Study 1 and 2. The effect was significant after removal of endpoint data, and all pairwise comparisons was significant (Sidak corrected, all *p* values < .001; see Fig 7).

**Fig 8:**
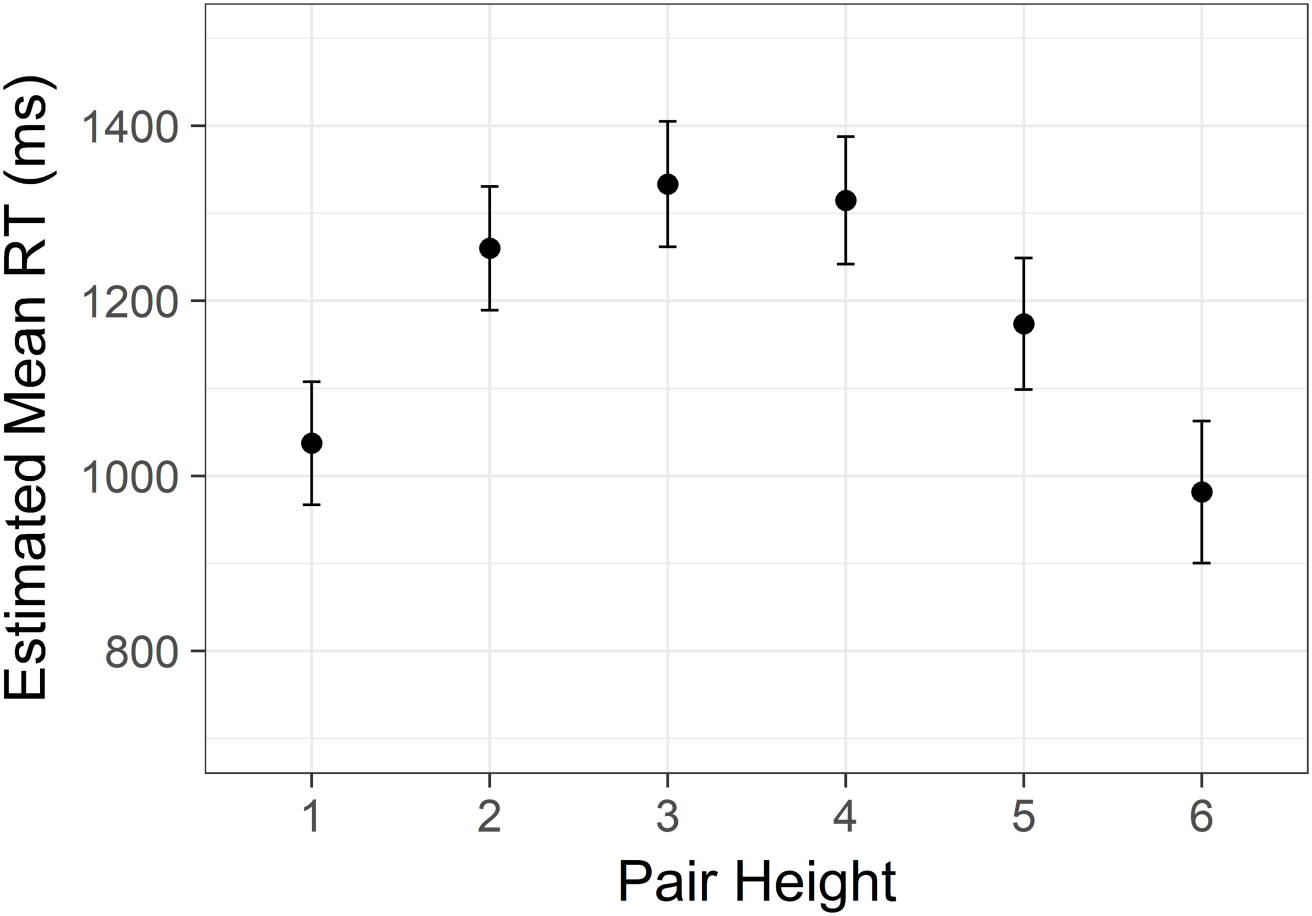
Non-linear size effect from Study 3. Estimated mean time spent correctly identifying the highest/lowest status target in a pair with increasing status of the lower member of the target pair. Y axis is scaled in milliseconds. Error bars represent 95% confidence intervals.

There was also a significant effect of height *F*(5,8338) = 203.97, *p* < .001, with estimated means again showing an inverted U-shape as in Studies 1 and 2 (Fig 8). This effect was also significant after removal of endpoints.

**Fig 9:**
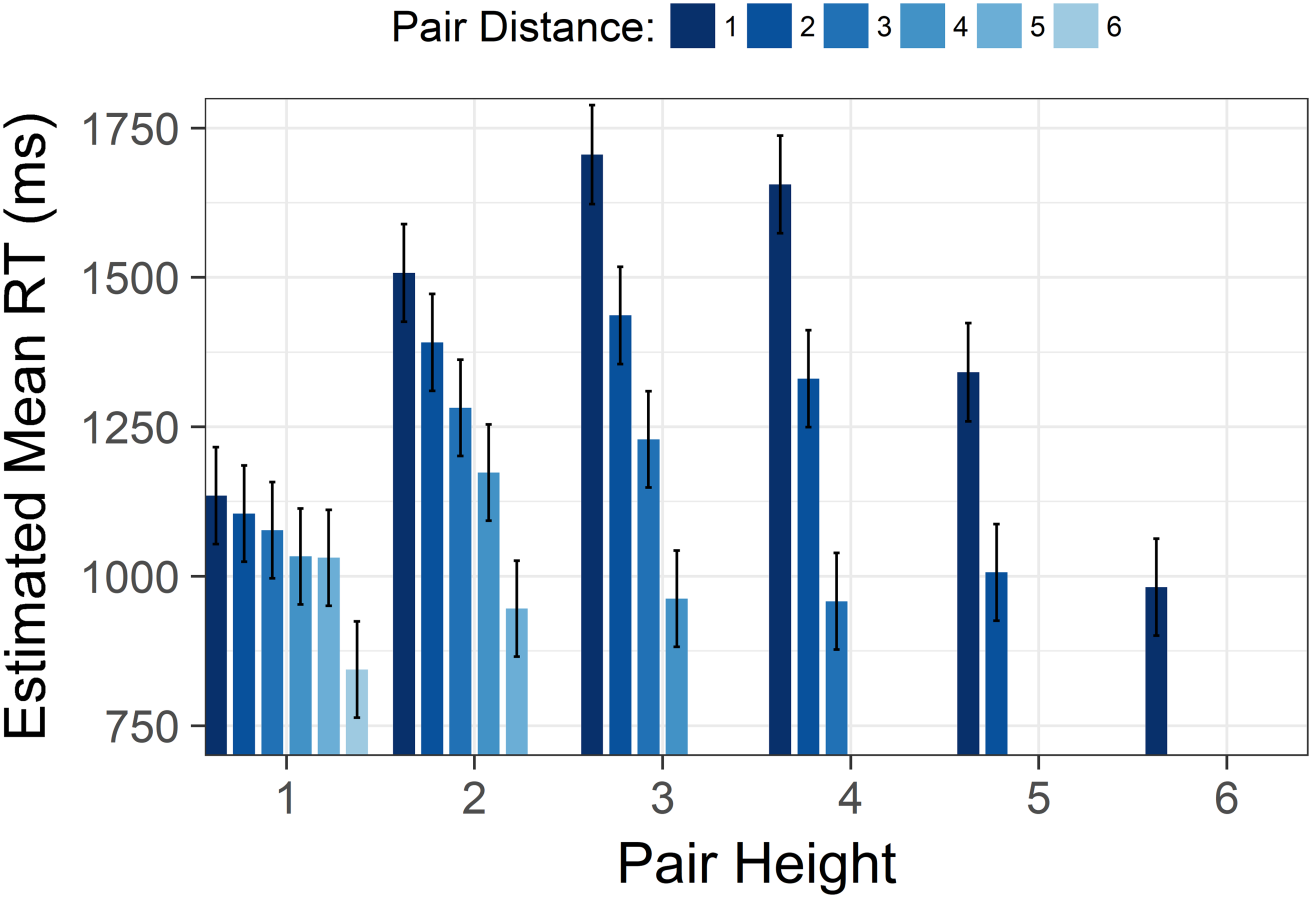
Size by distance interaction from Study 3. Estimated mean time spent correctly identifying the highest/lowest status target in a pair with increasing status of the lower member of the target pair. Each cluster displays the estimated means with increasing pair distance for that level of height. Y axis is scaled in milliseconds. Error bars represent 95% confidence intervals.

Finally, height and distance interacted significantly, *F*(10,8338) = 71.31, *p* < .001. In Study 3 we were able to meaningfully look at this interaction with end points excluded. The increase of the effect of distance with increasing levels of height was preserved, and the interaction was still significant *F*(3,3834) = 13.68, *p* < .001 (Fig 9).

As in the previous studies, several of the randomization and counterbalancing effects turned out to be significant while explaining only little variance. A full overview can be found in S1 Table.

### Discussion

Study 3 increased the number of levels in the hierarchies. The central findings of Studies 1 and 2 were replicated: the distance effect, an inverted U-shaped size effect, and the interaction all aligned with previous results. The larger number of levels also allowed the exclusion of trials including end-points while still retaining a meaningful range of values in the independent variables. Both the main effects of interest and the interaction between them was significant after the exclusion of end points. The inverted U-shape of the size effect also remained. We therefore conclude that the artificial processing advantage when judging end-points in our experimental task cannot alone account for the presence and shape of the size effect we observe.

## General discussion

The present three studies provide a very consistent picture of how comparisons of rank in social hierarchies are influenced by the level and distance in the hierarchy. First, the evidence successfully replicates and extends on earlier work on the distance effect. As hypothesized (H_1_), the participants responded faster with increasing status difference between the person or rank of each pair, confirming earlier work by von Hecker and colleagues [38], Ciao and colleagues [21,37], and Jiang and Zhu [39]. We are able to demonstrate this effect with higher resolution, and we observe a linear decrease of reaction time within hierarchies of both six or seven levels.

Importantly, the experimental paradigm used in this series of studies was designed to exclude potential confounds from earlier studies. Von Hecker and colleagues [38] presented their word pairs vertically, a stimulus presentation previously found to cue social power [43]. Chiao and colleagues [21,37] averaged rank differences to broad categories, giving a less granular account than the present results. Finally, Jiang and Zho [39] used group labels belonging to no particular social hierarchy, relying on separate power-ratings to make them directly comparable.

In addition to replicating the distance effect for social status, the results show a clear non-linear effect of the height of the pair (i.e., the size effect). Participants responded more quickly to pairs of lower and higher status as compared to intermediate status. This is different from the hypothesized effect (H_2_), where we expected that decisions starting from lower ranks in the hierarchy would be performed more quickly. Nevertheless, the effect of pair height was consistent across all three studies.

Considering the unexpected shape of the size effect, an experimental artefact was suspected of driving the effect in Study 1 and Study 2. We considered it possible that participants might adopt a strategy of immediately responding when seeing either extreme in a hierarchy, as there would be no need to make a comparison. Adding a seventh level to each hierarchy in Study 3 allowed us to exclude all trials including either extreme. The inverted U-shape persisted even when excluding these trials. We therefore reject this alternative explanation.

In the current work, we define pair height as the height of the lower member of the pair. A limitation of this definition is that a given height level includes pairs with varying distance levels. For that reason, it is essential to include the interaction term in the model, as we have done in the current analyses.

In the domain of numerosity in the AMS, definitions of size that take both height and distance into account have been proposed, such as the ratio of the two elements. However, this and other composite measures have been found to have low test-retest reliability [44]. Because the AMS is assumed to follow Weber’s law, a Weber’s fraction is sometimes calculated for each trial. The Weber’s fraction has a higher test-retest reliability than a simple ratio [44]. However, quantifying the distance between ranks in social hierarchies in order to calculate the Weber’s fraction is not as straight forward as with physical or numerical dimensions because no objective numbers are available to compare.

When plotting each distance level separately, the inverted U-shape is present for each distance level (see Figs 3, 6 and 9). Moreover, the inverted U-shape is evident for all distances where a sufficient number of height levels are possible (i.e. a height level range of three or more). We are therefore confident that the unexpected shape of the size effect is not driven by collapsing trials with varying distance levels into one height level.

What might cause the reverse U-shaped size effect? Whereas most physical magnitudes have a singular endpoint, social hierarchies have both an upper and a lower bound. The unexpected pattern could be explained as an increase in computational demand with increasing distance from either endpoint, resulting in the longest response times for intermediate ranks. One approach for testing this alternative hypothesis in future work would be to compare judgements of physical magnitudes with two endpoints.

However, this is not the only possible explanation. Unlike physical magnitudes, there is no objective measure of the distance between ranks in a social hierarchy. So far, all ranks within each hierarchy have been treated as equally spaced between the two endpoints. However, if the true spacing of the ranks are systematically skewed, so that intermediate ranks are perceived as more tightly spaced than the extremes, the apparent size effect could be an incidental byproduct of the distance effect. However, such a skew would have to manifest systematically across hierarchies, both novel and familiar, to explain the findings reported here.

Alternatively, it is possible that both high and low ranks are more salient, reflecting a high importance for an individual to preferentially attend to the extremes of a social hierarchy.

In addition, we consistently observed an interaction effect between the height and distance of the pairs, which was not hypothesized. With increasing social status, the effect of increasing distance became more pronounced. We currently cannot offer a post-hoc explanation for this result and consider it an exploratory finding. We realize this might be disappointing. However, scientific progress works by deriving hypotheses from theories and testing them. Falsification is informative. The size effect is derived from the theory that social status is represented as a magnitude, a notion that has been proposed by several contributions to the literature (see the Introduction). The fact that we do not find it as expected is a challenge to those theoretical notions, and they either must add new assumptions to the prevailing models or develop new theories. At the moment, abandoning the status-as-magnitude idea seems premature as the distance effect is quite clearly present.

In Study 2 and 3, participants made status judgements of ranks in already known social hierarchies. This process omits the typical ways humans infer status of other individuals. A broad psychological body of work shows that social status is conveyed and inferred through separate individual characteristics, such as physical size and facial features of masculinity and strength (for a review, see [45]). As such, the AMS’s involvement in status comparisons is independent from the process of inferring status from individual status cues, but rather result from the cognitive task of comparing agents or ranks along a mentally represented dimension. This is in line with research on numerosity in the AMS, where the assumption is that the distance and size effects should only occur in tasks in which the AMS is used [44].

Beyond visible characteristics, humans of course use a range of additional cultural signifiers and cues of power and status, most importantly wealth and education. In a long-lasting debate in sociological research, stratification of cultural consumption is seen either as a communication of economical class (homology argument) or a distinct social class (individualization arguments; for a review of this debate, see [46]). In addition, recent sociological survey-based research indicates that the variety rather than type of culture consumed signifies status; low-status individuals consume a limited range of culture (popular culture), whereas high-status individuals are cultural omnivores [46,47]. We would expect to find similar magnitude representation of all such cues to status.

Our results yield limited support for social status being represented in the AMS. As shown previously, the distance effect for social hierarchies manifests as predicted from physical dimensions in the magnitude system. Moreover, a size effect for social status was observed, although it is unclear at this time whether the effect is indicative of shared cognitive system with physical magnitudes. Importantly, the size effect is an interesting effect irrespective of the potential link to the AMS.

Nevertheless, the unexpected size effect and size by distance interaction highlights a limitation in the present series of studies. The hypothesis for the size effect was derived on the basis of an *a priori* mapping of social hierarchies to analogous physical dimensions, in which low status was defined as the endpoint from which the dimension stretched. In order to put forward clear, informed hypotheses about how the size effect should manifest if social status is treated as a magnitude in the AMS, a better understanding of the size effect in physical dimensions is required. Moreover, future studies should investigate alternative hypotheses for the size effect independent from the magnitude system.

In conclusion, we robustly demonstrate the distance effect for social status for both novel and well-learned hierarchies at a higher resolution and with more rigorous control of potential confounds than earlier work. Moreover, we demonstrate a novel shape of the size effect for social status, and an unexpected size by distance interaction. The findings provide both a confirmation and a challenge for the notion that social status is processed in the AMS.

## Supporting information

S1 Code Mixed model analysis SPSS syntax. (DOCX)

S1 Table Mixed model analysis SPSS output. (DOCX)
