# Supplementary material for: Evidence for magnitude representations of social hierarchies: Size and distance effects
Source: PLoS One. 2018 Sep 7;13(9):e0203263. doi: 10.1371/journal.pone.0203263 (PMC6128480; doi:10.1371/journal.pone.0203263)
Supplement: S1 Code — (DOCX) [file pone.0203263.s001.docx]

# Supporting information

# S1 Code. Mixed model analysis SPSS syntax.

This file contains in full the code (i.e. SPSS syntax) used to run the mixed model analysis for Study 1-3.

## Study 1

MIXED RTln BY Higher Pair_Distance PairHeightInverted BlockList.Sample BlockList

/CRITERIA=CIN(95) MXITER(100) MXSTEP(10) SCORING(1) SINGULAR(0.000000000001) HCONVERGE(0,

ABSOLUTE) LCONVERGE(0, ABSOLUTE) PCONVERGE(0.000001, ABSOLUTE)

/FIXED=

Higher

Pair_Distance

PairHeightInverted

BlockList.Sample

BlockList

Higher*Pair_Distance

Higher*PairHeightInverted

Higher*BlockList.Sample

Pair_Distance*PairHeightInverted

Pair_Distance*BlockList.Sample

PairHeightInverted*BlockList.Sample

Higher*BlockList

Pair_Distance*BlockList

PairHeightInverted*BlockList

BlockList.Sample*BlockList

Higher*Pair_Distance*PairHeightInverted

Higher*Pair_Distance*BlockList.Sample

Higher*PairHeightInverted*BlockList.Sample

Pair_Distance*PairHeightInverted*BlockList.Sample

Higher*Pair_Distance*BlockList

Higher*PairHeightInverted*BlockList

Higher*BlockList.Sample*BlockList

Pair_Distance*PairHeightInverted*BlockList

Pair_Distance*BlockList.Sample*BlockList

PairHeightInverted*BlockList.Sample*BlockList

Higher*Pair_Distance*PairHeightInverted*BlockList.Sample

Higher*Pair_Distance*PairHeightInverted*BlockList

Higher*Pair_Distance*BlockList.Sample*BlockList

Higher*PairHeightInverted*BlockList.Sample*BlockList

Pair_Distance*PairHeightInverted*BlockList.Sample*BlockList

Higher*Pair_Distance*PairHeightInverted*BlockList.Sample*BlockList

| SSTYPE(3)

/METHOD=REML

/PRINT=COVB SOLUTION TESTCOV

/RANDOM=INTERCEPT | SUBJECT(Subject) COVTYPE(VC)

/EMMEANS=TABLES(Higher) COMPARE ADJ(LSD)

/EMMEANS=TABLES(PairHeightInverted) COMPARE ADJ(LSD)

/EMMEANS=TABLES(Pair_Distance) COMPARE ADJ(sidak)

/EMMEANS=TABLES(PairHeightInverted*Pair_Distance) compare (PairHeightInverted) adj(lsd)

/EMMEANS=TABLES(PairHeightInverted*Pair_Distance) compare (Pair_Distance) adj(lsd).

## Study 2

MIXED RTln BY PickHigher PairHeight PairDist HierarchyOrder Hierarchy

/CRITERIA=CIN(95) MXITER(100) MXSTEP(10) SCORING(1) SINGULAR(0.000000000001) HCONVERGE(0,

ABSOLUTE) LCONVERGE(0, ABSOLUTE) PCONVERGE(0.000001, ABSOLUTE)

/FIXED=

PickHigher

PairHeight

PairDist

HierarchyOrder

Hierarchy

PickHigher*PairHeight

PickHigher*PairDist

PickHigher*HierarchyOrder

PickHigher*Hierarchy

PairHeight*PairDist

PairHeight*HierarchyOrder

PairHeight*Hierarchy

PairDist*HierarchyOrder

PairDist*Hierarchy

HierarchyOrder*Hierarchy

PickHigher*PairHeight*PairDist

PickHigher*PairHeight*HierarchyOrder

PickHigher*PairHeight*Hierarchy

PickHigher*PairDist*HierarchyOrder

PickHigher*PairDist*Hierarchy

PickHigher*HierarchyOrder*Hierarchy

PairHeight*PairDist*HierarchyOrder

PairHeight*PairDist*Hierarchy

PairHeight*HierarchyOrder*Hierarchy

PairDist*HierarchyOrder*Hierarchy

PickHigher*PairHeight*PairDist*HierarchyOrder

PickHigher*PairHeight*PairDist*Hierarchy

PickHigher*PairHeight*HierarchyOrder*Hierarchy

PickHigher*PairDist*HierarchyOrder*Hierarchy

PairHeight*PairDist*HierarchyOrder*Hierarchy

PickHigher*PairHeight*PairDist*HierarchyOrder*Hierarchy | SSTYPE(3)

/METHOD=REML

/PRINT=COVB SOLUTION TESTCOV

/RANDOM=INTERCEPT | SUBJECT(Subject) COVTYPE(VC)

/EMMEANS=TABLES(PickHigher) COMPARE ADJ(LSD)

/EMMEANS=TABLES(PairHeight) COMPARE ADJ(LSD)

/EMMEANS=TABLES(PairDist) COMPARE ADJ(sidak)

/EMMEANS=TABLES(PairHeight*PairDist) compare (pairheight) adj(lsd)

/EMMEANS=TABLES(PairHeight*PairDist) compare (pairdist) adj(lsd).

## Study 3

MIXED RTln BY PickHigher PairHeight PairDist HierarchyOrder Hierarchy

/CRITERIA=CIN(95) MXITER(100) MXSTEP(10) SCORING(1) SINGULAR(0.000000000001) HCONVERGE(0,

ABSOLUTE) LCONVERGE(0, ABSOLUTE) PCONVERGE(0.000001, ABSOLUTE)

/FIXED=

PickHigher

PairHeight

PairDist

HierarchyOrder

Hierarchy

PickHigher*PairHeight

PickHigher*PairDist

PickHigher*HierarchyOrder

PickHigher*Hierarchy

PairHeight*PairDist

PairHeight*HierarchyOrder

PairHeight*Hierarchy

PairDist*HierarchyOrder

PairDist*Hierarchy HierarchyOrder*Hierarchy

PickHigher*PairHeight*PairDist

PickHigher*PairHeight*HierarchyOrder

PickHigher*PairHeight*Hierarchy

PickHigher*PairDist*HierarchyOrder

PickHigher*PairDist*Hierarchy

PickHigher*HierarchyOrder*Hierarchy

PairHeight*PairDist*HierarchyOrder

PairHeight*PairDist*Hierarchy

PairHeight*HierarchyOrder*Hierarchy

PairDist*HierarchyOrder*Hierarchy

PickHigher*PairHeight*PairDist*HierarchyOrder

PickHigher*PairHeight*PairDist*Hierarchy

PickHigher*PairHeight*HierarchyOrder*Hierarchy

PickHigher*PairDist*HierarchyOrder*Hierarchy

PairHeight*PairDist*HierarchyOrder*Hierarchy

PickHigher*PairHeight*PairDist*HierarchyOrder*Hierarchy | SSTYPE(3)

/METHOD=REML

/PRINT=COVB SOLUTION TESTCOV

/RANDOM=INTERCEPT | SUBJECT(Subject) COVTYPE(VC)

/EMMEANS=TABLES(PickHigher) COMPARE ADJ(LSD)

/EMMEANS=TABLES(PairHeight) COMPARE ADJ(LSD)

/EMMEANS=TABLES(PairDist) COMPARE ADJ(sidak)

/EMMEANS=TABLES(PairHeight*PairDist) compare (pairheight) adj(lsd)

/EMMEANS=TABLES(PairHeight*PairDist) compare (pairdist) adj(lsd).
