# Supplementary material for: Evidence for magnitude representations of social hierarchies: Size and distance effects
Source: PLoS One. 2018 Sep 7;13(9):e0203263. doi: 10.1371/journal.pone.0203263 (PMC6128480; doi:10.1371/journal.pone.0203263)
Supplement: S1 Table — (DOCX) [file pone.0203263.s002.docx]

# Supporting information

# S1 Table. Mixed model analysis SPSS Output.

This file contains in full the fixed effects SPSS output from the mixed model analysis for Study 1-3.

## Study 1

Fixed effects table for dataset with trials containing hierarchy endpoints included.

| **Type III Tests of Fixed Effects^a^** | | | | |
| --- | --- | --- | --- | --- |
| Source | Numerator df | Denominator df | F | Sig. |
| Intercept | 1 | 27.895 | 30848.293 | .000 |
| Higher | 1 | 28.126 | .483 | .493 |
| Pair_Distance | 4 | 7426.284 | 197.789 | .000 |
| PairHeightInverted | 4 | 7426.609 | 100.069 | .000 |
| BlockList.Sample | 5 | 7440.015 | 4.600 | .000 |
| BlockList | 5 | 7448.953 | 3.939 | .001 |
| Higher * Pair_Distance | 4 | 7426.254 | .614 | .652 |
| Higher * PairHeightInverted | 4 | 7426.663 | 5.021 | .000 |
| Higher * BlockList.Sample | 5 | 7390.519 | 4.463 | .000 |
| Pair_Distance * PairHeightInverted | 6 | 7426.419 | 23.288 | .000 |
| Pair_Distance * BlockList.Sample | 20 | 7426.238 | .655 | .873 |
| PairHeightInverted * BlockList.Sample | 20 | 7426.631 | 1.242 | .208 |
| Higher * BlockList | 5 | 7415.958 | 7.542 | .000 |
| Pair_Distance * BlockList | 20 | 7426.233 | 1.408 | .106 |
| PairHeightInverted * BlockList | 20 | 7426.629 | 2.489 | .000 |
| BlockList.Sample * BlockList | 24 | 7315.107 | 5.003 | .000 |
| Higher * Pair_Distance * PairHeightInverted | 6 | 7426.481 | .251 | .959 |
| Higher * Pair_Distance * BlockList.Sample | 20 | 7426.232 | 2.086 | .003 |
| Higher * PairHeightInverted * BlockList.Sample | 20 | 7426.669 | 1.987 | .005 |
| Pair_Distance * PairHeightInverted * BlockList.Sample | 30 | 7426.417 | 1.341 | .101 |
| Higher * Pair_Distance * BlockList | 20 | 7426.239 | 1.316 | .156 |
| Higher * PairHeightInverted * BlockList | 20 | 7426.720 | 2.813 | .000 |
| Higher * BlockList.Sample * BlockList | 19 | 7289.714 | 3.408 | .000 |
| Pair_Distance * PairHeightInverted * BlockList | 30 | 7426.401 | 1.788 | .005 |
| Pair_Distance * BlockList.Sample * BlockList | 96 | 7426.253 | 1.448 | .003 |
| PairHeightInverted * BlockList.Sample * BlockList | 96 | 7426.624 | 3.040 | .000 |
| Higher * Pair_Distance * PairHeightInverted * BlockList.Sample | 30 | 7426.438 | .856 | .692 |
| Higher * Pair_Distance * PairHeightInverted * BlockList | 30 | 7426.443 | .797 | .776 |
| Higher * Pair_Distance * BlockList.Sample * BlockList | 76 | 7426.263 | 1.232 | .084 |
| Higher * PairHeightInverted * BlockList.Sample * BlockList | 75 | 7426.671 | 2.204 | .000 |
| Pair_Distance * PairHeightInverted * BlockList.Sample * BlockList | 144 | 7426.387 | 1.078 | .250 |
| Higher * Pair_Distance * PairHeightInverted * BlockList.Sample * BlockList | 109 | 7426.415 | 1.578 | .000 |
| a. Dependent Variable: rtln. | | | | |

Fixed effects table for dataset with trials containing hierarchy endpoints excluded.

| **Type III Tests of Fixed Effects^a^** | | | | |
| --- | --- | --- | --- | --- |
| Source | Numerator df | Denominator df | F | Sig. |
| Intercept | 1 | 27.951 | 30831.850 | .000 |
| Higher | 1 | 28.456 | .533 | .471 |
| Pair_Distance | 2 | 2824.651 | 73.778 | .000 |
| PairHeightInverted | 2 | 2825.891 | 24.098 | .000 |
| BlockList.Sample | 5 | 2838.466 | 4.068 | .001 |
| BlockList | 5 | 2846.870 | 5.683 | .000 |
| Higher * Pair_Distance | 2 | 2824.858 | 1.167 | .312 |
| Higher * PairHeightInverted | 2 | 2826.144 | 2.337 | .097 |
| Higher * BlockList.Sample | 5 | 2812.584 | 8.007 | .000 |
| Pair_Distance * PairHeightInverted | 1 | 2825.211 | .067 | .795 |
| Pair_Distance * BlockList.Sample | 10 | 2824.637 | .868 | .563 |
| PairHeightInverted * BlockList.Sample | 10 | 2825.983 | 1.352 | .197 |
| Higher * BlockList | 5 | 2833.059 | 4.790 | .000 |
| Pair_Distance * BlockList | 10 | 2824.579 | 2.314 | .010 |
| PairHeightInverted * BlockList | 10 | 2825.707 | 3.479 | .000 |
| BlockList.Sample * BlockList | 24 | 2764.509 | 3.866 | .000 |
| Higher * Pair_Distance * PairHeightInverted | 1 | 2825.534 | 1.038 | .308 |
| Higher * Pair_Distance * BlockList.Sample | 10 | 2824.741 | 2.845 | .002 |
| Higher * PairHeightInverted * BlockList.Sample | 10 | 2825.885 | 1.508 | .130 |
| Pair_Distance * PairHeightInverted * BlockList.Sample | 5 | 2824.631 | .663 | .651 |
| Higher * Pair_Distance * BlockList | 10 | 2824.829 | 1.873 | .044 |
| Higher * PairHeightInverted * BlockList | 10 | 2826.181 | 1.305 | .222 |
| Higher * BlockList.Sample * BlockList | 19 | 2743.982 | 4.712 | .000 |
| Pair_Distance * PairHeightInverted * BlockList | 5 | 2824.569 | .793 | .554 |
| Pair_Distance * BlockList.Sample * BlockList | 48 | 2824.527 | 1.491 | .016 |
| PairHeightInverted * BlockList.Sample * BlockList | 48 | 2825.654 | 2.483 | .000 |
| Higher * Pair_Distance * PairHeightInverted * BlockList.Sample | 5 | 2824.594 | .948 | .449 |
| Higher * Pair_Distance * PairHeightInverted * BlockList | 5 | 2824.647 | .961 | .440 |
| Higher * Pair_Distance * BlockList.Sample * BlockList | 38 | 2824.627 | 1.568 | .015 |
| Higher * PairHeightInverted * BlockList.Sample * BlockList | 37 | 2825.882 | 1.424 | .047 |
| Pair_Distance * PairHeightInverted * BlockList.Sample * BlockList | 24 | 2824.577 | 1.158 | .270 |
| Higher * Pair_Distance * PairHeightInverted * BlockList.Sample * BlockList | 15 | 2824.907 | 1.407 | .134 |
| a. Dependent Variable: rtln. | | | | |

## Study 2

Fixed effects table for dataset with trials containing hierarchy endpoints included.

| **Type III Tests of Fixed Effects^a^** | | | | |
| --- | --- | --- | --- | --- |
| Source | Numerator df | Denominator df | F | Sig. |
| Intercept | 1 | 27.348 | 56709.864 | .000 |
| PickHigher | 1 | 27.348 | 2.894 | .100 |
| PairHeight | 4 | 4525.065 | 110.750 | .000 |
| PairDist | 4 | 4525.028 | 150.390 | .000 |
| HierarchyOrder | 2 | 4548.077 | 14.076 | .000 |
| Hierarchy | 2 | 4548.158 | 66.241 | .000 |
| PickHigher * PairHeight | 4 | 4525.065 | 78.813 | .000 |
| PickHigher * PairDist | 4 | 4525.028 | 6.297 | .000 |
| PickHigher * HierarchyOrder | 2 | 4548.077 | 6.701 | .001 |
| PickHigher * Hierarchy | 2 | 4548.158 | 9.113 | .000 |
| PairHeight * PairDist | 6 | 4525.039 | 52.655 | .000 |
| PairHeight * HierarchyOrder | 8 | 4525.090 | 1.746 | .083 |
| PairHeight * Hierarchy | 8 | 4525.094 | 4.494 | .000 |
| PairDist * HierarchyOrder | 8 | 4525.027 | .981 | .448 |
| PairDist * Hierarchy | 8 | 4525.019 | 2.252 | .021 |
| HierarchyOrder * Hierarchy | 4 | 4436.249 | 10.070 | .000 |
| PickHigher * PairHeight * PairDist | 6 | 4525.039 | 1.796 | .096 |
| PickHigher * PairHeight * HierarchyOrder | 8 | 4525.090 | 2.899 | .003 |
| PickHigher * PairHeight * Hierarchy | 8 | 4525.094 | 4.569 | .000 |
| PickHigher * PairDist * HierarchyOrder | 8 | 4525.027 | .565 | .808 |
| PickHigher * PairDist * Hierarchy | 8 | 4525.019 | .530 | .835 |
| PickHigher * HierarchyOrder * Hierarchy | 4 | 4436.249 | 7.815 | .000 |
| PairHeight * PairDist * HierarchyOrder | 12 | 4525.034 | 1.412 | .152 |
| PairHeight * PairDist * Hierarchy | 12 | 4525.041 | 5.433 | .000 |
| PairHeight * HierarchyOrder * Hierarchy | 16 | 4525.096 | 2.805 | .000 |
| PairDist * HierarchyOrder * Hierarchy | 16 | 4525.032 | 1.257 | .216 |
| PickHigher * PairHeight * PairDist * HierarchyOrder | 12 | 4525.034 | 1.614 | .081 |
| PickHigher * PairHeight * PairDist * Hierarchy | 12 | 4525.041 | 2.335 | .006 |
| PickHigher * PairHeight * HierarchyOrder * Hierarchy | 16 | 4525.096 | 1.982 | .011 |
| PickHigher * PairDist * HierarchyOrder * Hierarchy | 16 | 4525.032 | 1.171 | .283 |
| PairHeight * PairDist * HierarchyOrder * Hierarchy | 24 | 4525.052 | 1.489 | .059 |
| PickHigher * PairHeight * PairDist * HierarchyOrder * Hierarchy | 24 | 4525.052 | 1.124 | .306 |
| a. Dependent Variable: RTln. | | | | |

Fixed effects table for dataset with trials containing hierarchy endpoints excluded.

| **Type III Tests of Fixed Effects^a^** | | | | |
| --- | --- | --- | --- | --- |
| Source | Numerator df | Denominator df | F | Sig. |
| Intercept | 1 | 27.389 | 39663.842 | .000 |
| PickHigher | 1 | 27.389 | 4.267 | .048 |
| PairHeight | 2 | 1726.238 | 9.174 | .000 |
| PairDist | 2 | 1726.114 | 50.758 | .000 |
| HierarchyOrder | 2 | 1746.351 | 7.558 | .001 |
| Hierarchy | 2 | 1746.150 | 3.971 | .019 |
| PickHigher * PairHeight | 2 | 1726.238 | 16.034 | .000 |
| PickHigher * PairDist | 2 | 1726.114 | 1.685 | .186 |
| PickHigher * HierarchyOrder | 2 | 1746.351 | 9.675 | .000 |
| PickHigher * Hierarchy | 2 | 1746.150 | 6.717 | .001 |
| PairHeight * PairDist | 1 | 1726.154 | .364 | .546 |
| PairHeight * HierarchyOrder | 4 | 1726.188 | .450 | .772 |
| PairHeight * Hierarchy | 4 | 1726.250 | 3.244 | .012 |
| PairDist * HierarchyOrder | 4 | 1726.078 | .638 | .635 |
| PairDist * Hierarchy | 4 | 1726.080 | 1.404 | .230 |
| HierarchyOrder * Hierarchy | 4 | 1697.178 | 4.593 | .001 |
| PickHigher * PairHeight * PairDist | 1 | 1726.154 | 5.804 | .016 |
| PickHigher * PairHeight * HierarchyOrder | 4 | 1726.188 | 1.626 | .165 |
| PickHigher * PairHeight * Hierarchy | 4 | 1726.250 | 6.882 | .000 |
| PickHigher * PairDist * HierarchyOrder | 4 | 1726.078 | 1.160 | .327 |
| PickHigher * PairDist * Hierarchy | 4 | 1726.080 | 1.726 | .141 |
| PickHigher * HierarchyOrder * Hierarchy | 4 | 1697.178 | 6.372 | .000 |
| PairHeight * PairDist * HierarchyOrder | 2 | 1726.199 | 1.571 | .208 |
| PairHeight * PairDist * Hierarchy | 2 | 1726.194 | 8.435 | .000 |
| PairHeight * HierarchyOrder * Hierarchy | 8 | 1726.283 | 3.411 | .001 |
| PairDist * HierarchyOrder * Hierarchy | 8 | 1726.119 | 1.477 | .161 |
| PickHigher * PairHeight * PairDist * HierarchyOrder | 2 | 1726.199 | .589 | .555 |
| PickHigher * PairHeight * PairDist * Hierarchy | 2 | 1726.194 | 1.836 | .160 |
| PickHigher * PairHeight * HierarchyOrder * Hierarchy | 8 | 1726.283 | 1.159 | .321 |
| PickHigher * PairDist * HierarchyOrder * Hierarchy | 8 | 1726.119 | 1.063 | .386 |
| PairHeight * PairDist * HierarchyOrder * Hierarchy | 4 | 1726.271 | 1.278 | .277 |
| PickHigher * PairHeight * PairDist * HierarchyOrder * Hierarchy | 4 | 1726.271 | .359 | .838 |
| a. Dependent Variable: RTln. | | | | |

## Study 3

Fixed effects table for dataset with trials containing hierarchy endpoints included.

| **Type III Tests of Fixed Effects^a^** | | | | |
| --- | --- | --- | --- | --- |
| Source | Numerator df | Denominator df | F | Sig. |
| Intercept | 1 | 50.697 | 78568.434 | .000 |
| PickHigher | 1 | 50.697 | 11.138 | .002 |
| PairHeight | 5 | 8338.199 | 203.965 | .000 |
| PairDist | 5 | 8338.112 | 306.021 | .000 |
| HierarchyOrder | 1 | 8341.228 | 20.904 | .000 |
| Hierarchy | 1 | 8341.228 | 155.853 | .000 |
| PickHigher * PairHeight | 5 | 8338.199 | 79.471 | .000 |
| PickHigher * PairDist | 5 | 8338.112 | 23.052 | .000 |
| PickHigher * HierarchyOrder | 1 | 8341.228 | 6.369 | .012 |
| PickHigher * Hierarchy | 1 | 8341.228 | .108 | .743 |
| PairHeight * PairDist | 10 | 8338.170 | 71.309 | .000 |
| PairHeight * HierarchyOrder | 5 | 8338.224 | 4.643 | .000 |
| PairHeight * Hierarchy | 5 | 8338.224 | 6.296 | .000 |
| PairDist * HierarchyOrder | 5 | 8338.097 | .738 | .595 |
| PairDist * Hierarchy | 5 | 8338.097 | 1.504 | .185 |
| HierarchyOrder * Hierarchy | 1 | 50.697 | .428 | .516 |
| PickHigher * PairHeight * PairDist | 10 | 8338.170 | 2.023 | .027 |
| PickHigher * PairHeight * HierarchyOrder | 5 | 8338.224 | 1.377 | .230 |
| PickHigher * PairHeight * Hierarchy | 5 | 8338.224 | .791 | .556 |
| PickHigher * PairDist * HierarchyOrder | 5 | 8338.097 | .283 | .923 |
| PickHigher * PairDist * Hierarchy | 5 | 8338.097 | .943 | .452 |
| PickHigher * HierarchyOrder * Hierarchy | 1 | 50.697 | 3.626 | .063 |
| PairHeight * PairDist * HierarchyOrder | 10 | 8338.169 | .349 | .967 |
| PairHeight * PairDist * Hierarchy | 10 | 8338.169 | 5.493 | .000 |
| PairHeight * HierarchyOrder * Hierarchy | 5 | 8338.199 | 1.267 | .275 |
| PairDist * HierarchyOrder * Hierarchy | 5 | 8338.112 | 1.792 | .111 |
| PickHigher * PairHeight * PairDist * HierarchyOrder | 10 | 8338.169 | 2.172 | .017 |
| PickHigher * PairHeight * PairDist * Hierarchy | 10 | 8338.169 | 2.169 | .017 |
| PickHigher * PairHeight * HierarchyOrder * Hierarchy | 5 | 8338.199 | 1.918 | .088 |
| PickHigher * PairDist * HierarchyOrder * Hierarchy | 5 | 8338.112 | 1.368 | .233 |
| PairHeight * PairDist * HierarchyOrder * Hierarchy | 10 | 8338.170 | .489 | .898 |
| PickHigher * PairHeight * PairDist * HierarchyOrder * Hierarchy | 10 | 8338.170 | 1.592 | .102 |
| a. Dependent Variable: rtln. | | | | |

Fixed effects table for dataset with trials containing hierarchy endpoints excluded.

| **Type III Tests of Fixed Effects^a^** | | | | |
| --- | --- | --- | --- | --- |
| Source | Numerator df | Denominator df | F | Sig. |
| Intercept | 1 | 50.894 | 69345.878 | .000 |
| PickHigher | 1 | 50.894 | 6.050 | .017 |
| PairHeight | 3 | 3833.721 | 36.493 | .000 |
| PairDist | 3 | 3833.310 | 108.099 | .000 |
| HierarchyOrder | 1 | 3836.767 | 20.948 | .000 |
| Hierarchy | 1 | 3836.767 | 13.941 | .000 |
| PickHigher * PairHeight | 3 | 3833.721 | 27.258 | .000 |
| PickHigher * PairDist | 3 | 3833.310 | 5.108 | .002 |
| PickHigher * HierarchyOrder | 1 | 3836.767 | 1.127 | .289 |
| PickHigher * Hierarchy | 1 | 3836.767 | .294 | .587 |
| PairHeight * PairDist | 3 | 3833.525 | 13.683 | .000 |
| PairHeight * HierarchyOrder | 3 | 3833.852 | 3.542 | .014 |
| PairHeight * Hierarchy | 3 | 3833.852 | 14.143 | .000 |
| PairDist * HierarchyOrder | 3 | 3833.357 | .150 | .930 |
| PairDist * Hierarchy | 3 | 3833.357 | 6.746 | .000 |
| HierarchyOrder * Hierarchy | 1 | 50.894 | .004 | .952 |
| PickHigher * PairHeight * PairDist | 3 | 3833.525 | .546 | .651 |
| PickHigher * PairHeight * HierarchyOrder | 3 | 3833.852 | 1.998 | .112 |
| PickHigher * PairHeight * Hierarchy | 3 | 3833.852 | 2.876 | .035 |
| PickHigher * PairDist * HierarchyOrder | 3 | 3833.357 | .950 | .416 |
| PickHigher * PairDist * Hierarchy | 3 | 3833.357 | 1.536 | .203 |
| PickHigher * HierarchyOrder * Hierarchy | 1 | 50.894 | 3.111 | .084 |
| PairHeight * PairDist * HierarchyOrder | 3 | 3833.540 | .211 | .889 |
| PairHeight * PairDist * Hierarchy | 3 | 3833.540 | .845 | .469 |
| PairHeight * HierarchyOrder * Hierarchy | 3 | 3833.721 | .388 | .762 |
| PairDist * HierarchyOrder * Hierarchy | 3 | 3833.310 | .366 | .778 |
| PickHigher * PairHeight * PairDist * HierarchyOrder | 3 | 3833.540 | 3.902 | .009 |
| PickHigher * PairHeight * PairDist * Hierarchy | 3 | 3833.540 | 1.682 | .169 |
| PickHigher * PairHeight * HierarchyOrder * Hierarchy | 3 | 3833.721 | 1.120 | .340 |
| PickHigher * PairDist * HierarchyOrder * Hierarchy | 3 | 3833.310 | .994 | .395 |
| PairHeight * PairDist * HierarchyOrder * Hierarchy | 3 | 3833.525 | .368 | .776 |
| PickHigher * PairHeight * PairDist * HierarchyOrder * Hierarchy | 3 | 3833.525 | 1.540 | .202 |
| a. Dependent Variable: rtln. | | | | |
